# Pregnancy-Related Complications and Incidence of Atrial Fibrillation: A Systematic Review

**DOI:** 10.3390/jcm12041316

**Published:** 2023-02-07

**Authors:** Tariq Al Bahhawi, Abdulwahab Aqeeli, Stephanie L. Harrison, Deirdre A. Lane, Flemming Skjøth, Iain Buchan, Andrew Sharp, Nathalie Auger, Gregory Y. H. Lip

**Affiliations:** 1 Liverpool Centre for Cardiovascular Science at University of Liverpool, Liverpool John Moores University, Liverpool Heart and Chest Hospital, Liverpool L14 3PE, UK; 2 Department of Cardiovascular and Metabolic Medicine, Institute of Life Course and Medical Sciences, University of Liverpool, Liverpool L7 8TX, UK; 3 Faculty of Medicine, Jazan University, Jazan 82817, Saudi Arabia; 4 Joint Program of Preventive Medicine, Saudi Commission for Health Specialties, Jeddah 21589, Saudi Arabia; 5 Department of Clinical Medicine, Faculty of Health, Aalborg University, DK-9100 Aalborg, Denmark; 6 Unit of Clinical Biostatistics, Aalborg University Hospital, DK-9100 Aalborg, Denmark; 7 Department of Public Health, Policy and Systems, Institute of Population Health, University of Liverpool, Liverpool L69 3GF, UK; 8 Harris-Wellbeing Preterm Birth Research Centre, University of Liverpool, Liverpool L8 7SS, UK; 9 Liverpool Women’s Hospital NHS Foundation Trust, Liverpool L8 7SS, UK; 10 University of Montreal Hospital Research Centre, School of Public Health, University of Montreal, Montreal, QC H2X 0A9, Canada; 11 Department of Epidemiology, Biostatistics and Occupational Health, McGill University, Montreal, QC H3A 1G1, Canada

**Keywords:** atrial fibrillation, cardiovascular disease, pregnancy complications, hypertensive disorders of pregnancy, pre-eclampsia

## Abstract

Pregnancy-related complications are associated with a higher risk of various incident cardiovascular diseases, but their specific potential relationship with incident atrial fibrillation (AF) is less clear. This systematic review summarises the available evidence from observational studies which have examined associations between pregnancy-related complications and the risk of AF. MEDLINE and EMBASE (Ovid) were searched for studies between 1990 to 10 February 2022. Pregnancy-related complications examined included hypertensive disorders of pregnancy (HDP), gestational diabetes, placental abruption, preterm birth, small-for-gestational-age and stillbirth. Study selection, data extraction and quality assessment were completed independently by two reviewers. Narrative synthesis was used to evaluate the results of the included studies. Nine observational studies were included, with eight eligible for narrative synthesis. Sample sizes ranged from 1839 to 2,359,386. Median follow-up ranged from 2 to 36 years. Six studies reported that pregnancy-related complications were associated with a significantly increased risk of incident AF. Hazard ratios (HRs) (95% confidence intervals) for the four studies that evaluated HDP ranged from 1.1 (0.8–1.6) to 1.9 (1.4–2.7). For the four studies that evaluated pre-eclampsia, HRs ranged from 1.2 (0.9–1.6) to 1.9 (1.7–2.2). Current evidence from observational studies suggests pregnancy-related complications are associated with a significantly higher risk of incident AF. However, only a small number of studies examining each pregnancy-related complication were identified, and considerable statistical heterogeneity was observed. Further large-scale prospective studies are required to confirm the association between pregnancy-related complications and incident AF.

## 1. Introduction

Pregnancy-related complications such as hypertensive disorders of pregnancy (HDP), preterm birth and fetal growth restriction are interrelated disorders that share risk factors with atrial fibrillation (AF) [1]. Women experiencing pregnancy-related complications are at higher risk of developing AF-related risk factors such as hypertension, diabetes, renal dysfunction, dyslipidaemia and cardiovascular diseases (CVD) compared to women without pregnancy-related complications [2]. Globally, the prevalence of HDP is approximately 10% of all pregnancies [3]. Similarly, the global preterm birth rate has been estimated at 10.6% [4], whereas hyperglycaemia may impact 16.9% of pregnancies [5]. Previous systematic reviews of observational studies have shown that pre-eclampsia and gestational hypertension are associated with a two- to four-fold increased risk of CVD [6,7,8,9,10]. Gestational diabetes nearly doubles the risk of CVD [10,11,12]. Preterm birth has been associated with a two-fold greater risk of developing maternal CVD [13,14].

AF is the most common sustained cardiac arrhythmia and is estimated to impact approximately 33.5 million people globally [15]. Recently, the European Society of Cardiology re-estimated the lifetime risk of AF to be one in three individuals among European populations aged ≥ 55 years [16]. While the incidence and prevalence of AF are higher among males, females with AF have greater morbidity, including a higher risk of stroke [17].

AF and pregnancy-related complications share common pathophysiological mechanisms and risk factors. The incidence of later-life chronic hypertension is increased up to four-fold among women with HDP compared to those with normotensive pregnancies. [6,8] Hypertension is an important risk factor for incident AF [18]. Moreover, pregnancy-related complications are associated with cardiac remodelling and dysfunction, which can persist beyond the postpartum period [19,20,21,22]. These shared common pathophysiological mechanisms and risk factors could help target the prevention, earlier identification and treatment of women at higher risk of developing AF, helping to reduce sex disparities in AF morbidity.

The aim of this systematic review was to evaluate the available evidence examining the association between pregnancy-related complications and incident AF.

## 2. Methods

The reporting of this systematic review was based on the Preferred Reporting Items for Systematic Reviews and Meta-Analyses (PRISMA) guidelines and Synthesis Without Meta-analysis (SWiM) in systematic reviews: reporting guideline [23,24]. The full PRISMA checklist table is reported in the Supplementary Material (Table S1). The protocol was registered on PROSPERO (reference number: CRD42020180058).

### 2.1. Search Strategy

Ovid MEDLINE(R) and Ovid EMBASE were searched to identify eligible studies from 1990 to 10 February 2022. The full search details are described in the Supplementary Material (Tables S2 and S3). The search strategy combined pregnancy-related complications, cardiovascular diseases and AF. In addition, AF keywords with wild cards were used to capture wording variation. 

The search was restricted to human studies, articles published in English and observational study designs, including cross-sectional, case-control, and cohort studies.

Studies identified through the searches were imported to EndNote (version X9) and duplicate records were removed. De-duplicated results were exported to Rayyan QCRI for screening [25]. Two reviewers (TA and AA) independently screened the titles and abstracts according to the inclusion/exclusion criteria. The full-texts of articles identified as potentially relevant at the title and abstract stage were retrieved in full-text and assessed independently by two reviewers (TA and AA). Disagreements were resolved through discussion and consulting with co-authors (SLH and DAL). Reference lists of included studies and relevant reviews were also screened to identify any additional relevant articles.

### 2.2. Inclusion and Exclusion Criteria

Observational studies including cross-sectional, case-control and cohort studies which examined associations between pregnancy-related complications and incidence of AF after delivery were eligible for inclusion. Any study which included women who had a diagnosis of AF prior to their first pregnancy or women who developed AF during their index pregnancy or labour period was excluded. Authors of studies that reported AF as part of composite CVD were contacted to provide relevant data regarding AF separately. Reviews, conference posters or abstracts, editorials and commentaries were excluded.

### 2.3. Data Extraction

Data were extracted independently by two reviewers (TA and AA) from the selected articles using a pre-prepared standardised data extraction form and any disagreements were resolved by discussion. The data were extracted into a spreadsheet collating: article information including author names, publication year, country, funding sources and conflicts of interest; population and comparator data including exclusion criteria, age, co-morbidities, prevalence and type of all pregnancy-related complications reported; methodology including methods used to identify AF, study period, type of exposure, follow-up time, list of variables adjusted for in analyses; results including incidence of AF among exposed and non-exposed, and adjusted and unadjusted point estimates.

### 2.4. Quality Assessment

Two reviewers (TA and AA) independently assessed the risk of bias in the included studies using the Newcastle–Ottawa Scale (NOS) [26].

### 2.5. Statistical Analysis

Meta-analysis was not conducted due to heterogeneity in the included studies, such as variation in exposure definitions, age of the cohorts and follow-up time. Therefore, a narrative synthesis using summarised effect estimates was conducted to describe the evidence.

## 3. Results

### 3.1. Search Results

**Figure 1:**
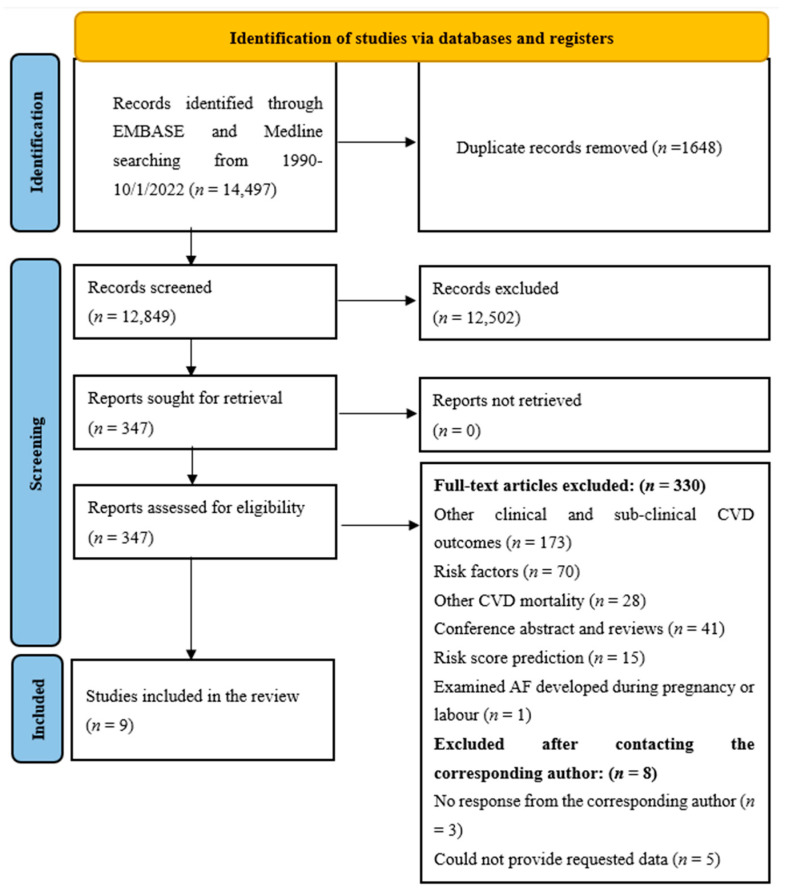
PRISMA flow diagram of study inclusion.

The searches identified 14,497 articles from MEDLINE and EMBASE (Figure 1). After removing duplicates (*n* = 1648), 12,849 titles and abstracts were screened. Of these, 12,502 (97.3%) were excluded, and 347 full-text articles were retrieved and screened. Following full-text screening, nine studies met the inclusion criteria [27,28,29,30,31,32,33,34,35]. Of the nine included studies, one study [32] had a case–control design, and eight studies [27,28,29,30,31,33,34,35] were cohort studies, of which one study utilised data from a prospective cohort, [29] and seven used longitudinal data from electronic health records. [27,28,30,31,33,34,35] One of the included studies provided unpublished data (after contacting the author) on the incidence rate, median follow-up time and hazard ratios for preterm birth and pre-eclampsia with incident AF [27]. The narrative synthesis described eight unique cohorts. Two studies [28,32] reported data for the same cohort, and the data from the cohort [28] was included in the narrative synthesis, as this provided more detailed relevant data.

### 3.2. Study Characteristics

#### 3.2.1. Participants

The number of participants included in the eight cohort studies ranged from 1839 [28] to 2,359,386 [34]. All studies were published from 2012 onwards, with seven [27,28,29,30,33,34,35] published since 2019. Characteristics of the included studies are presented in Table 1.

#### 3.2.2. Data Sources

The included studies utilised data from Canada [27,31], the USA [28,32], Denmark, [33] Korea [35] and the UK [29,30,34]. The two studies conducted in Canada used routinely collected health data from Ontario [31] and Quebec [27]. Both studies carried out in the USA used the same cohort from the Rochester Epidemiology Project [28,32], while the studies conducted in Denmark [33] and Korea [35] utilised routinely collected data at a nationwide level. The studies conducted in the UK used the UK Biobank data [29] and data from CALIBER [30]. 

#### 3.2.3. Exposures

Five studies examined single exposures [28,29,30,33,35]. Of these, four studies examined HDP [28,29,30,35], and one study examined gestational diabetes [33]. Three studies examined multiple exposures: one study examined pre-eclampsia and gestational hypertension [34]. One examined preterm birth and pre-eclampsia [27], and one examined the composite exposure of various pregnancy complications, including HDP, placental abruption or infarction, intrauterine fetal death or preterm birth [31]. Seven studies used either the International Classification of Diseases (ICD)-8, (ICD)-9 or ICD-10 to identify the exposure [27,29,30,31,33,34,35], with one study incorporating other codes, such as Read codes [30]. Two studies used an algorithm incorporating parameters such as blood pressure readings during pregnancy to define HDP (Table 1) [28,32].

#### 3.2.4. Outcomes

All the included studies used either (ICD)-8, ICD-9 or ICD-10 codes to identify AF. [27,28,29,30,31,32,33,34,35] Additionally, some studies included other coding systems, such as the Mayo-adapted Hospital International Classification of Diseases Adapted (HICDA) codes [32] or Clinical Classification Codes [28]. All cohort studies included incident AF shortly after delivery [27,28,30,31,33,34,35] except one which included incident AF after the UK Biobank baseline visit (2006–2010) [29]. Further details of the definitions of the exposures and AF are summarised in Table 1.

#### 3.2.5. Follow-Up

Follow-up durations ranged from a median of 2 [34] to 36 years [28] postpartum. Four studies had a median follow-up time of 2–9.25 years [29,30,31,34], while three studies had median follow-ups of 16 years [27,33] and 36 years [28] (Table 2).

#### 3.2.6. Adjustment for Potential Confounding Factors

Six studies adjusted for factors such as socioeconomic status, maternal age and diabetes [27,30,31,33,34,35]. Two studies adjusted for a smaller group of risk factors: the first adjusted for age at enrolment and race [29], and the second adjusted for education, smoking and obesity [28]. Table 2 summarises the risk factors adjusted for each individual study.

#### 3.2.7. Baseline Characteristics

The baseline characteristics for the participants in the eight included studies [27,28,29,30,31,33,34,35] are presented in Table S4; only six studies reported baseline characteristics [29,30,31,33,34,35]. The participants in studies that reported ethnicity were predominantly white. [29,30,33,34] Women with pregnancy-related complications compared to those without pregnancy-related complications tended to have a higher prevalence of hypertension [29,30,31,35], diabetes [29,30,31,34,35], multifetal birth [30,31,35] and obesity [29,30,31,33,35].

### 3.3. Assessment of Study Quality

All included studies were rated as good quality according to the Newcastle–Ottawa Scale, with total scores ranging from 6–9 (Table 1 and Table S5).

#### Pregnancy-Related Complications and Incident Atrial Fibrillation

Six of the eight included studies reported a significant association between pregnancy-related complications and incident AF [27,28,30,31,33,35]. The associations were attenuated but remained statistically significant after adjustment for potential confounders (Table 2). Two studies reported no association between pregnancy complications and incident AF [29,34]. The event rate for incident AF among women with pregnancy-related complications was in the range of 0.1%–1% for all cohort studies [27,29,30,31] except for one study, which was 38.0% [28].

### 3.4. Hypertensive Disorders of Pregnancy

Four studies examined the association between any HDP and incident AF [28,29,30,35]. Two studies defined HDP as gestational hypertension, pre-eclampsia, eclampsia, superimposed pre-eclampsia and pre-existing hypertension during pregnancy [28,30]. one study defined HDP as gestational hypertension, pre-eclampsia, eclampsia and HELLP syndrome [29] and one study used gestational hypertension, pre-eclampsia and eclampsia (Table 2) [35].

**Figure A1:**
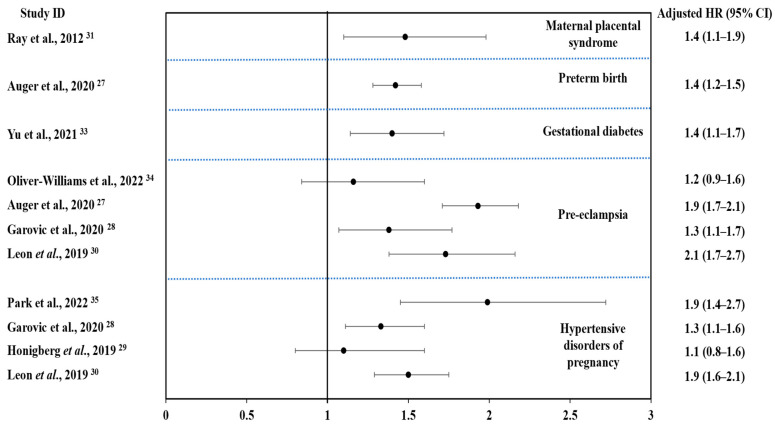
Forest plot summarising the adjusted hazard ratios between pregnancy complications and incident atrial fibrillation by the pregnancy complication sub-type.

Three studies (*n* = 3,340,888) reported an increased risk of incident AF during follow-up among women exposed to HDP, with HRs (95% confidence interval) ranging from: 1.3 (1.1–1.6) to 1.9 (1.4–2.7) (Table 2) [28,30,35]. One study did not observe a statistically significant association between HDP and incident AF, HR 1.1 (0.8–1.6) (Table 2 and Figure A1) [29].

### 3.5. Pre-Eclampsia

Three of four studies that evaluated pre-eclampsia reported a positive association between pre-eclampsia and incident AF [27,28,30], with HRs ranging from 1.4 (1.1–1.8) to 1.9 (1.7–2.2). One study reported no association between pre-eclampsia and incident AF, HR 1.2 (0.9–1.6) (see Table 2 and Figure A1) [34].

### 3.6. Preterm Birth

One study examined the association between preterm birth and incident AF [27]. The risk of developing AF among women who had a previous preterm birth was significantly higher than among women who had not experienced a preterm birth, with HR 1.4 (1.3–1.6) (see Table 2 and Figure A1) [27].

### 3.7. Gestational Diabetes

One study evaluated the role of gestational diabetes and incident AF and revealed a higher risk of AF among women with gestational diabetes compared to women without gestational diabetes, with HR 1.4 (1.1–1.7) (see Table 2 and Figure A1) [33].

### 3.8. Composite Pregnancy Complications

One study examined the association of composite pregnancy complications such as maternal placental syndrome and incident AF, reporting a higher risk of incident AF in women with a history of maternal placental syndrome who had a higher risk of incident AF compared to women without HR 1.5 (1.1–2.0) (see Table 2 and Figure A1) [31].

## 4. Discussion

This systematic review identified eight cohort studies [27,28,29,30,31,33,34,35] and one case–control study [32] examining the association between pregnancy-related complications and incident AF. Overall, our findings indicate that pregnancy complications, particularly pre-eclampsia, may be associated with an increased risk of incident AF; however, there was considerable heterogeneity between studies, which may have resulted from inconsistency in the definition of the pregnancy-related complications and/or variation in the follow-up time. Moreover, there are other potential explanations for this heterogeneity, such as timing of data collection due to changes in pregnancy care, exposure definition, medical advances and postpartum follow-up. Therefore, these results need to be interpreted with caution.

Previous systematic reviews have investigated the association between pregnancy-related complications and CVD, with most focusing on either a composite of CVD outcomes, myocardial infarction, stroke, heart failure or CVD mortality with pre-eclampsia [6,7,8,9], gestational diabetes [11,12], preterm birth [13,14,36], miscarriage [37] and various pregnancy-related complications. [10] Further, one review evaluated the incidence of AF during pregnancy. [38] Our review extends and clarifies this existing knowledge by focusing on associations between pregnancy-related complications and incident AF after pregnancy.

Our findings, which showed an overall increased risk of AF, were in line with previous reviews. [6,9,10,12,13] A recent review of reviews found a significant association between pre-eclampsia and a two-fold increased risk of ischemic heart disease and a composite CVD outcome, a four-fold increased risk for heart failure and a 1.5–1.9-fold increased risk of stroke [39].

There were some differences in the methods used to identify AF between the studies, particularly between countries. For example, in the UK, read codes are used by general practitioners to capture primary care transactions with greater detail/completeness of clinical coding than most other parts of the world. Further, undetected or incorrectly diagnosed AF may abate the validity of the assessment of the outcome. This is demonstrated in one nested case–control study where 147 incident AF cases were identified using ICD-9 and Mayo-adapted HICDA codes, but after a chart review, 36 cases were excluded, and 2 cases were added [32]. Furthermore, one study enrolled women from the UK biobank with previous live birth history at the baseline visit (2006–2010) [29]. The documentation of incident AF was during follow-up from the baseline visit, while the birth history could have been up to 30 years before the baseline visit [29]. The reported mean age at enrolment was approximately 52 years for women with HDP compared to 57 years for women without HDP [29]. The time from first birth to baseline visit was 23 years for women with HDP and 31 years and women without HDP. This gap between birth and UK biobank baseline visits creates an immortal time interval because women who developed incident AF in that period were counted as having prevalent AF by design and excluded.

The event rate for incident AF among women with pregnancy-related complications in Garovic et al. [28] was 38% compared to around 1% for all other cohort studies [27,29,30,31]. The higher incidence of AF reported by Garovic et al. [28] is likely due to a longer follow-up time, with a median of 36 years. This highlights the importance of longer follow-up times in evaluating incident AF. Furthermore, Ray et al. [31] found the mean age of onset of AF, heart failure or ventricular dysrhythmia was 38 years, while the mean age at delivery was 29 years. Additionally, the divergence in the cumulative probability of AF hospitalisation increased with time [31]. Therefore, it is possible that studies with shorter follow-up times or studies that include younger women may underestimate incident AF as these women may not have had enough time to develop AF. Hence, these results indicate that studies with longer follow-up times (>10 years) are preferable when examining the association between pregnancy-related complications and incident AF.

Regarding baseline characteristics such as hypertension and diabetes, there was variability in the definition and inclusion of these conditions between studies which led to large variability in the estimated prevalence between studies. It has commonly been assumed that higher cardiovascular risk during or after pregnancy among women with pregnancy-related complications is largely due to shared pre-pregnancy cardiovascular risk factors [40,41]. However, higher cardiovascular risk profiles later in life in part explain the association between pregnancy-related complications and CVD [42,43].

Peripartum cardiomyopathy is an uncommon form of cardiomyopathy that develops during the later stages of pregnancy or shortly after giving birth [44]. In a matched retrospective cohort, AF was one of the most common incident CVD among women with peripartum cardiomyopathy one year after delivery [45]. Moreover, previous systematic reviews have reported that the prevalence of pregnancy-related complications, such as HDP was 22% (95% CI; 16%–28%) [46] and the prevalence of AF was 5% (1%–11%) [47] among women with peripartum cardiomyopathy. However, a recent study showed that pregnancy-related complications were associated with higher risk of CVD independently from peripartum cardiomyopathy [48]. Another recent study that utilised data from the National Inpatient Sample database, highlighted that women with peripartum cardiomyopathy and AF had a higher rate of in-hospital mortality (4% vs. 0.7%, *p* = 0.02) and greater use of mechanical ventilation (14% vs. 6.7%, *p* = 0.044), when compared to those without AF [49]. These findings emphasise the significance of early detection and management of AF in women with peripartum cardiomyopathy in order to improve their outcomes and reduce healthcare resource utilisation [49]. 

A recent review highlighted that numerous guidelines have recognised HDP as a predictor of CVD [50]. Indeed, the 2019 International Federation of Gynaecology and Obstetrics suggested that pregnancy-related complications, including HDP, gestational diabetes mellitus, foetal growth restriction, preterm birth, recurrent pregnancy loss and placental abruption, be acknowledged as predictors of CVD [51]. However, the recent 2020 European Society of Cardiology (ESC) guidelines for the diagnosis and management of AF did not highlight the effect of pregnancy-related complications on the risk of incident AF [16]. This may be due to the small number of previously published studies that have evaluated the association between pregnancy-related complications and incident AF, as demonstrated in this review.

The potential association between pregnancy-related complications and incident AF likely involves multiple mechanisms. First, risk factors such as metabolic syndrome, which are common to multiple outcomes, may drive incident AF [52]. Second, cardiac remodelling and fibrosis, which occur with pregnancy-related complications, can lead to long-term impaired cardiac function [22,53,54,55] and increase the risk of incident AF. Another possible explanation may be that pregnancy-related complications increase the risk of incident AF risk factors, such as hypertension, an unfavourable lipid profile and diabetes [56,57,58]. Finally, genetic factors may play a role in the observed associations, as a recent study found that single nucleotide polymorphisms (SNP) rs2200733 near the PITX2 gene was associated with pre-eclampsia and AF [59].

### Strengths and Limitations

This review has several strengths. First, a comprehensive search strategy was used and included CVD in general as an outcome rather than limiting the searches to studies of AF alone. The review methodology was comprehensive, with screening and data extraction independently conducted by two reviewers. However, there are some limitations. First, only nine studies were eligible for inclusion. Second, the exposure definitions varied between studies which could increase the heterogeneity between studies. Third, some of the studies could not control for common confounding factors due to insufficient recording in routine healthcare record data sources. Fourth, the follow-up time varied from less than 1 year to 38 years, and only three studies had an adequate long-term follow-up; hence, the incidence of AF was low. Fifth, most studies included young pregnant women (<30 years old) with short follow-up duration; therefore, the risk of incident AF may be underestimated. Sixth, AF misclassification is possible resulting from utilising codes to identify AF. Seventh, the inability to determine whether the increased incidence of AF is related to the spontaneous onset of labour or iatrogenic preterm delivery among women who have experienced preterm birth. Finally, of the nine included studies, eight were from Western countries, which may limit the generalisability of the findings.

Large-scale, prospective cohort studies with long-term follow-up (>10 years), are needed to expose the relationship between pregnancy-related complications and incident AF. Future studies should also aim to investigate the temporal relationships between pregnancy-related complications, AF and peripartum cardiomyopathy.

## 5. Conclusions

Pregnancy-related complications (pre-eclampsia, hypertensive disorders of pregnancy, preterm birth and maternal placental syndrome) could be associated with a higher risk of incident AF. However, the small number of included studies and the significant heterogeneity in those studies suggest that further research is required.

## Figures and Tables

**Table 1:** Characteristics of the included studies examining pregnancy-related complications and incidence of atrial fibrillation.

First Author, Year, Country	Study Design, No. of Participants, Timeframe for Data Collected	Data Sources	Participant Selection Criteria	Study Exposure (s), Definition (s)	Study Outcome(s), Definition (s)
Ray et al., 2012 [31] Canada	Cohort study, 1,130,764 1992–2009.	Routinely collected healthcare administrative databases for Ontario health insurance plan	Inclusion: Aged 14–50 years at the time of delivery with ≥ 20 weeks gestation Exclusion: ≥1 of the following conditions occurring < 365 days before the date of delivery: Cardiac dysrhythmia HF Pericardial, endocardial or myocardial disease, cardiomyopathy or peripartum cardiomyopathy or rheumatic or valvular heart disease Congenital heart disease CAD Cerebrovascular disease PAD Thyroid disease	MPS included any of the following: Gestational hypertension Pre-eclampsia or eclampsia Severe pre-eclampsia Placental abruption Placental infarction Intrauterine fetal death Poor fetal growth Preterm delivery < 37 weeks gestation MPS defined by ICD-9 and ICD-10-CA codes during index delivery hospitalisation	Atrial fibrillation or flutter Ventricular tachycardia or fibrillation HF Defined using ICD-9 and ICD-10-CA codes > 365 days post-delivery
Scantlebury et al., 2018 [32] USA	Nested case–control, 105 case 105 control, 1976–2012 Enrolment: Delivery during 1976–1982.	Rochester Epidemiology Project contains information about 7566 women who gave birth to a live or stillborn infant in Olmsted County, USA, from 1976–1982	All women with sufficient pregnancy information. Exclusion: Previous AF diagnosis before 1st pregnancy. Previous HTN diagnosis before 1st pregnancyใ No research authorisation (In the sensitivity analysis, women who had other structural heart disease or rheumatic or other valvular disease and their matched pairs were excluded)	Any form of HDP which included: Gestational hypertension Chronic hypertension Pre-eclampsia Pre-eclampsia superimposed on chronic hypertension Eclampsia in any pregnancy Exposures were defined by diagnostic algorithms designed by the research team	Atrial fibrillation AF or atrial flutter diagnosis using ICD-9 codes and Mayo-adapted HICDA codes and confirmed manually from the patient medical chart.
Leon et al., 2019 [30] UK	Cohort, 1,303,365 The study cohort included 1,899,150 unique pregnancies 1997–2016.	CALIBER resource, which combined routinely collected data from CPRD, HES and ONS	All women aged between 11 to 49 years with > 20 weeks’ gestation consented to data linkage. Exclusion: Records outside the eligibility period Duplicate records In the analyses, any incident of a single or composite outcome of interest that occurred within 6 weeks from delivery was excluded	Pre-eclampsia/eclampsia with or without preterm birth. HDP included: gestational hypertension, pre-eclampsia, superimposed pre-eclampsia, or pre-existing hypertension during pregnancy Pre-eclampsia was defined as any diagnosis recorded using Read or ICD-10 codes within 20 weeks before or after the pregnancy end date. HDP was defined as any diagnosis recorded using Read or ICD10 Codes for the previously mentioned conditions. Preterm was defined as any pregnancy record ending before 37 weeks gestation.	Atrial fibrillation Ischemic stroke Intracerebral haemorrhage Subarachnoid haemorrhage Stroke not otherwise specified MI Stable angina Unstable angina Coronary heart disease not otherwise specified PAD Abdominal aortic aneurysm HF Diagnosis for any previously mentioned conditions based on CALIBER EHR algorithms using Read or ICD-10 codes.
Honigberg et al., 2019 [29] UK	Cohort 220,024 2006–2016. Enrolment: 2006–2010.	UK biobank, which is a prospective cohort, recruited > 500,000 individuals aged from 40 to 69 years during 2006–2010	All women in the UK Biobank who reported one or more live birth. Exclusion: Women with congenital heart disease Nulliparous women at bassline No parity data The analysis excluded the following condition independently: Prevalence of any CVD Prevalent CAD Any prevalent ASCVD (CAD, ischemic stroke, or peripheral artery disease) Women with HF Women with less than one-year follow-up Women with cancer	HDP includes: Gestational hypertension. Pre-eclampsia. Eclampsia. HELLP syndrome. HDP was defined as any diagnosis using an ICD code or self-reported at recruitment	Atrial fibrillation or flutter CAD HF Aortic stenosis Mitral regurgitation Ischemic stroke PAD Venous thromboembolism Diagnosis for any previously mentioned conditions based on ICD-9&10 codes from inpatient and outpatient records, death registrations, and primary care diagnoses
Garovic et al., 2020 [28] USA	Cohort, 1839 1976–2017. Enrolment: Delivery during 1976–1982.	Rochester Epidemiology Project contains information about women who gave birth to a live or stillborn infant in Olmsted County, USA, from 1976 -1982	Inclusion: Lived in Olmsted County when she had her first pregnancy ≥ 20 weeks’ gestation between 1976–1982 Resident of Olmsted County at least 75% of the time between her first pregnancy and the time when she underwent a hysterectomy, died or had her 46th birthday (whichever came first), according to the REP timeline. Resident of Olmsted County when she underwent a hysterectomy, died or had her 46th birthday (whichever came first) For all of her pregnancies, adequate information has been reported Exclusion: No consent to the use of their medical records for research Insufficient pregnancy information reported	HDP includes: Gestational hypertension. Pre-eclampsia. Eclampsia. Superimposed pre-eclampsia. Pre-existing hypertension. HDP was defined by a validated electronic diagnostic algorithm and using HICDA codes.	Cardiac arrhythmias (AF) CAD Congestive HF Stroke Chronic kidney disease Dementia Diagnosis using ICD-9 and Clinical Classification Codes.
Auger et al., 2020 [27] Canada	Cohort, 1,199,364 1989–2017.	Discharge abstracts in the Maintenance and Use of Data for the Study of Hospital Clientele registry in Quebec, Canada	Inclusion: All participants with delivery histories during the study period Exclusion: Pre-existing outcome Death during the index delivery. Invalid health insurance numbers	Preterm delivery birth at <37 completed weeks of gestation, based on ultrasound estimates from the first or second trimester. Pre-eclampsia was defined as any diagnosis by ICD-9 and ICD-10-CA codes	Hospitalisations from: MI. Ischemic stroke. Other cardiovascular disorders. AF based on unpublished data provided by the author. Defined using ICD-9 and ICD-10.
Yu et al., 2021 [33] Denmark	Cohort, 1,002,486 1978–2016.	Multiple Danish registers: Danish Civil Registration System, Danish Medical Birth Registry, Danish National Patient Register, Danish Register of Causes of Death, and Danish Integrated Database for Longitudinal Labour Market Research	Inclusion: All women had their first pregnancy during 1978–2016 Exclusion: Age less than 18 Pre-existing diabetes Pre-existing CVD Pre-existing congenital heart disease Pre-existing cancer	Gestational diabetes Defined using ICD-8 and ICD-10.	Ischemic heart disease Myocardial infarction Cerebrovascular disease Stroke Heart failure Atrial fibrillation Hypertensive disease Deep vein thrombosis Pulmonary embolism Peripheral artery disease Coronary artery bypass graft (CABG) Percutaneous coronary intervention (PCI) Other CVDs Defined by ICD-8, ICD-10 and procedure/surgery codes.
Park et al., 2022 [35] Korea	Cohort study, 2,035,684 2007–2015.	Korean National Health Insurance Database	Inclusion: All women gave birth between 2007–2015 and had 1 year of medical records before pregnancy Exclusion: Arrhythmia 1 year prior to or during pregnancy Pre-existing hypertension Previous pre-eclampsia	HDP includes: Gestational hypertension. Pre-eclampsia. Eclampsia Defined by ICD-10	Lethal arrhythmias Atrial flutter or fibrillation Atrioventricular block Paroxysmal tachycardia Premature beats Right bundle-branch block Defined by ICD-10
Oliver-Williams et al., 2022 [34] UK	Cohort study, 2,359,386 1997–2015	Hospital Episode Statistics (HES) database UK	Inclusion: All women give birth to at least one singleton live birth between 1997–2015 Exclusion Multifetal birth History of CVD History of hypertension	Gestational hypertension Pre-eclampsia Defined by ICD-10	CAD Angina MI HF Cardiomyopathy Atrial Fibrillation and flutter Ventricular arrhythmias, cardiac arrest and sudden cardiac death Stroke PAD Abdominal aortic aneurysm Defined by ICD-10

AF: Atrial Fibrillation; ASCVD: Atherosclerotic Cardiovascular Disease; CAD: Coronary Artery Disease; CALIBER: cardiovascular disease research using linked bespoke studies and electronic health records; CPRD: Clinical Practice Research Datalink; HDP: Hypertensive disorder of pregnancy; HELLP syndrome: Hemolysis, elevated liver enzyme levels, and low platelet levels; HER: Electronic Health Record; HES: Hospital Episode Statistics; HF: Heart Failure; HICDA: Hospital International Classification of Diseases Adapted; HTN: hypertension; ICD: International Classification of Diseases; MI: Myocardial infarction; MPS: Maternal Placental Syndrome; OSN: Office for National Statistics; PAD: Peripheral Artery Disease; SD: Standard deviation.

**Table 2:** Results of observational studies examining pregnancy-related complications and incidence of atrial fibrillation.

First Author, Year, Country	Maternal Age at Index Pregnancy, Mean (SD)	Follow-Up, Median (IQR) Duration	Incidence or Prevalence, *n* (%)	AF Event Rate, *n* (%)	Crude Risk Estimate of Association with AF (and 95% CI)	Adjusted Measure of The Association (and 95% CI)	Factors Adjusted for
Ray et al., 2012 [31] Canada	MPS = 29.7 (5.8) Non-MPS = 29.4 (5.5)	MPS = 7.8 (3.5 to 12.0) years Non-MPS = 7.8 (3.5 to 12.3) years	Incidence Incidence rate per 10,000 person years: MPS group = 0.87 Non-MPS group = 0.50 Prevalence Not reported	MPS group 51/57,242 = 0.09% Non-MPS group 488/1,055,522 = 0.05%	HR 1.76 (1.32–2.36)	HR 1.48 (1.10–1.98)	Socioeconomic quintile, rural residence, maternal age, length of stay in the index delivery hospital, diabetes mellitus, obesity, coronary artery disease, dyslipidaemia, multiple gestations, thyroid disease and drug dependence or tobacco use
Scantlebury et al., 2018 [32] USA	Age at index date (AF diagnosis): Cases = 56.56 (8.01) Control = 56.36 (7.71)	Years between first pregnancy and index date mean (SD) Cases = 32.11 (8.11) Control = 31.40 (7.59)	Not reported	Not applicable	HPD OR 2.60 (1.21–6.04) Pre-eclampsia OR 1.83 (0.62–6.04)	HPD OR 2.12 (0.92–5.23) Pre-eclampsia OR 1.20 (0.37–4.21)	Hypertension at the time of index date and body mass index > 30 kg/m^2^ at the first prenatal visit
Leon et al., 2019 [30] UK	Pre-eclampsia 28.61 (6.29) No Pre-eclampsia 28.47 (6.15)	Overall median (IQR) = 9.25 (5.53–13.78)	Not reported	Pre-eclampsia 86/25,554 = 0.34% HDP 228/109,500 = 0.21% Pre-eclampsia group with preterm 10/6868 = 0.01%	Pre-eclampsia HR 2.19 (1.76–2.72) HDP HR 1.9 (1.65–2.18) Pre-eclampsia with preterm HR 3.14 (1.69–5.85)	Pre-eclampsia HR 1.73 (1.38–2.16) HDP HR1.5 (1.29–1.75) Pre-eclampsia with preterm HR 1.98 (1.06–3.72)	Index of multiple deprivations, maternal ethnicity, maternal age, diabetes before pregnancy, hypertension before pregnancy and a cluster term to account for correlation between pregnancies within a single woman
Honigberg et al., 2019 [29] UK	Mean age (SD) at enrolment. HDP 52.3 (8.7) Non HDP 57.4 (7.8)	Median (IQR) = 7 (6.3 to 7.7) years, Overall range: 0 to 10 years	Incidence 3115/218,117= 1.4% Prevalence 5022/220,024 = 2.3%	HDP 29/2795 = 1.04% Non HDP 3086/215,322 = 1.43%	Not reported	HDP HR 1.1 (0.8–1.6)	Age at enrolment and race
Garovic et al., 2020 [28] USA	Not reported	HDP median (IQR) 36.2 years (23.5–38.2) No HDP 35.8 years (13.7–37.9)	Incidence (per 10,000 person years) 529/40,643 = 130 Prevalence Not reported	HDP 214/563 = 38.01% Non HDP 315/1138 = 27.68% Pre-eclampsia 110/293 = 37.50% Non pre-eclampsia 163/595 = 27.39%	HDP HR 1.35 (1.13–1.61) Pre-eclampsia HR 1.37 (1.08–1.75)	HDP: HR 1.33 (1.11–1.60) Pre-eclampsia HR 1.38 (1.07–1.77)	Education, smoking and obesity
Auger et al., 2020 [27] Canada	Not reported	Median is 16.7 years	Incidence (per 10,000 person years) Pre-eclampsia: 2.4 Preterm 1.8 Prevalence Not reported	Pre-eclampsia 286/69,360 = 0.41% Preterm 419/127,297 = 0.33%	Not reported	Pre-eclampsia HR 1.93 (1.71–2.18) ^a^ Preterm HR 1.42 (1.28–1.58) ^a^ Preterm birth or Pre-eclampsia: HR 1.61 (1.47–1.76) ^a^	Socioeconomic deprivation, maternal age, parity, multiple births, comorbidity (obesity, type 1 or 2 diabetes mellitus, dyslipidaemia, and alcohol, tobacco, or substance use) and year of delivery
Yu et al., 2021 [33] Denmark	Median age at the first delivery = 27 years, IQR (24–30 years)	Median follow-up time = 16.2 years, IQR (7.7–25.4) years	Incidence (per 1000 person years) Gestational diabetes group = 0.38 Non-gestational diabetes group = 0.46	Gestational diabetes 75/21,353 = 0.35%	HR 1.68 (1.37–2.06)	HR 1.40 (1.14–1.72)	First delivery time period, age at first delivery, parity education, smoking during pregnancy, obesity, cohabitation, residence, country of origin, maternal history of CVD and paternal CVD history
Park et al., 2022 [35] Korea	Mean age (SD) at birth. HDP 31.63 (3.86) Non HDP 31.12 (3.48)	Not reported	Not reported	Not reported	1-year HR 2.45 (2.07–2.90) Any time after delivery up to 7 years Not reported	1-year 2.27 (1.91–2.69) Any time after delivery up to 7 years HR 1.99 (1.45–2.72)	Age, primipara, caesarean section, multifetal pregnancy, body mass index, systolic and diastolic blood pressures, fasting blood glucose, aspartate aminotransferase levels, alanine aminotransferase levels, total cholesterol levels and current smoking
Oliver-Williams et al., 2022 [34] UK	Mean age (SD) at birth. Gestational hypertension 27.53 (5.74) Pre-eclampsia 27.31 (5.85) Non HDP 26.85 (5.80)	median follow up = 2.3 years 5th–95th percentiles = 0.3–12.1	Not reported	Not reported	Not reported	Gestational hypertension 1.20 (0.94–1.54) Pre-eclampsia 1.25 (0.96–1.62)	Maternal age at delivery, socioeconomic status, ethnicity and diabetes

AF: Atrial Fibrillation; CI: confidence interval; HDP: Hypertensive disorder of pregnancy; HR: Hazard ratio; IQR: Inter-quartile range; MPS: Maternal Placental Syndrome; N: Number; OR: Odd ratio; PE: Pre-eclampsia; SD: Standard deviation. ^a^ unpublished data.

## Data Availability

The data used in this article are available in the article and in its online supplementary material.

## Supplementary Materials

The following are available online at https://www.mdpi.com/article/10.3390/jcm12041316/s1, Table S1: PRISMA checklist; Table S2: Search strategy for Ovid MEDLINE(R) from 1990 to April 06, 2020, and updated on 10 February 2022; Table S3: Search strategy for Ovid Embase from 1990 to April 06, 2020, and updated on 10 February 2022; Table S4: Baseline characteristics of the participants in the included studies and Table S5: Risk of Bias Assessment in included Studies using Newcastle-Ottawa Scale.
