# Supplementary material for: Pregnancy-Related Complications and Incidence of Atrial Fibrillation: A Systematic Review
Source: J Clin Med. 2023 Feb 7;12(4):1316. doi: 10.3390/jcm12041316 (PMC9959873; doi:10.3390/jcm12041316)
Supplement: Supplementary file 1 [file jcm-12-01316-s001.zip › jcm-2111511-SI.pdf]

SUPPLEMENTAL MATERIAL

Table S1. PRISMA checklist.

Section/topic	#	Checklist item	Reported on page #
**TITLE**			
Title	1	Identify the report as a systematic review, meta-analysis, or both.	Title page
**ABSTRACT**			
Structured summary	2	Provide a structured summary including, as applicable: background; objectives; data sources; study eligibility criteria, participants, and interventions; study appraisal and synthesis methods; results; limitations; conclusions and implications of key findings; systematic review registration number.	1-2
**INTRODUCTION**			
Rationale	3	Describe the rationale for the review in the context of what is already known.	3-4
Objectives	4	Provide an explicit statement of questions being addressed with reference to participants, interventions, comparisons, outcomes, and study design (PICOS).	4
**METHODS**			
Protocol and registration	5	Indicate if a review protocol exists, if and where it can be accessed (e.g., Web address), and, if available, provide registration information including registration number.	4
Eligibility criteria	6	Specify study characteristics (e.g., PICOS, length of follow-up) and report characteristics (e.g., years considered, language, publication status) used as criteria for eligibility, giving rationale.	4-5
Information sources	7	Describe all information sources (e.g., databases with dates of coverage, contact with study authors to identify additional studies) in the search and date last searched.	4-5
Search	8	Present full electronic search strategy for at least one database, including any limits used, such that it could be repeated.	4-5 and Table S2&3 (Supplemental Material)
Study selection	9	State the process for selecting studies (i.e., screening, eligibility, included in systematic review, and, if applicable, included in the meta-analysis).	5 and figure1

Data collection process	10	Describe method of data extraction from reports (e.g., piloted forms, independently, in duplicate) and any processes for obtaining and confirming data from investigators.	6
Data items	11	List and define all variables for which data were sought (e.g., PICOS, funding sources) and any assumptions and simplifications made.	6
Risk of bias in individual studies	12	Describe methods used for assessing risk of bias of individual studies (including specification of whether this was done at the study or outcome level), and how this information is to be used in any data synthesis.	6
Summary measures	13	State the principal summary measures (e.g., risk ratio, difference in means).	6-7
Synthesis of results	14	Describe the methods of handling data and combining results of studies, if done, including measures of consistency (e.g., I^2) for each meta-analysis.	7
Risk of bias across studies	15	Specify any assessment of risk of bias that may affect the cumulative evidence (e.g., publication bias, selective reporting within studies).	7
Additional analyses	16	Describe methods of additional analyses (e.g., sensitivity or subgroup analyses, meta-regression), if done, indicating which were pre-specified.	Figure S1&2 (Supplemental Material)
**RESULTS**			
Study selection	17	Give numbers of studies screened, assessed for eligibility, and included in the review, with reasons for exclusions at each stage, ideally with a flow diagram.	7 and figure1
Study characteristics	18	For each study, present characteristics for which data were extracted (e.g., study size, PICOS, follow-up period) and provide the citations.	8-9 and table 1&2
Risk of bias within studies	19	Present data on risk of bias of each study and, if available, any outcome level assessment (see item 12).	Table S4
Results of individual studies	20	For all outcomes considered (benefits or harms), present, for each study: (a) simple summary data for each intervention group (b) effect estimates and confidence intervals, ideally with a forest plot.	Table 2
Synthesis of results	21	Present results of each meta-analysis done, including confidence intervals and measures of consistency.	10-11

Risk of bias across studies	22	Present results of any assessment of risk of bias across studies (see Item 15).	Table S4
Additional analysis	23	Give results of additional analyses, if done (e.g., sensitivity or subgroup analyses, meta-regression [see Item 16]).	Figure S1&2 (Supplemental Material)
**DISCUSSION**			
Summary of evidence	24	Summarize the main findings including the strength of evidence for each main outcome; consider their relevance to key groups (e.g., healthcare providers, users, and policy makers).	11-15
Limitations	25	Discuss limitations at study and outcome level (e.g., risk of bias), and at review-level (e.g., incomplete retrieval of identified research, reporting bias).	15
Conclusions	26	Provide a general interpretation of the results in the context of other evidence, and implications for future research.	15-16
**FUNDING**			
Funding	27	Describe sources of funding for the systematic review and other support (e.g., supply of data); role of funders for the systematic review.	17

Table S2: Search strategy for Ovid MEDLINE(R) from 1990 to April 06, 2020, and updated on 10 February 2022

N	Search Terms	No. Of Identified Records
1	exp pregnancy complications/	421289
2	exp Hypertension, Pregnancy-Induced/	36094
3	exp Diabetes, Gestational/	12215
4	exp Abortion, Spontaneous/	34845
5	exp infant, low birth weight/	33863
6	exp infant, premature/	54947
7	exp Infant, Small for Gestational Age/	7226
8	exp Premature Birth/	13388
9	exp stillbirth/	4746
10	exp Fetal Death/	29154
11	exp Abruptio Placentae/	2238
12	exp Fetal Growth Retardation/	16133
13	exp cardiovascular disease/	2354946
14	exp heart diseases/	1113336
15	exp Atrial Fibrillation/	54072
16	(arrhythmi* or cardiac dysrhythmia* or atrial fibrillation or afib or a-fib or atrial flutter* or bradycardi* or heart block* or	293611

	tachycardi* or Tachyarrhythmi* or ventricular fibrillation* or ventricular flutter*).ab,hw,kf,kw,ti.	
17	Epidemiologic studies/	8261
18	exp case control studies/	1068210
19	exp cohort studies/	1975645
20	Case control.tw.	123168
21	(cohort adj (study or studies)).tw.	199305
22	Cohort analy$.tw.	7817
23	(Follow up adj (study or studies)).tw.	48722
24	(observational adj (study or studies)).tw.	103410
25	Longitudinal.tw.	239927
26	Retrospective.tw.	516331
27	Cross sectional.tw.	342651
28	Cross-sectional studies/	323190
29	1 or 2 or 3 or 4 or 5 or 6 or 7 or 8 or 9 or 10 or 11 or 12	482610
30	13 or 14 or 15 or 16	2420458
31	17 or 18 or 19 or 20 or 21 or 22 or 23 or 24 or 25 or 26 or 27 or 28	2955620
32	29 and 30 and 31	11533
33	limit 32 to (english language and humans and yr="1990 - Current")	9708
34	limit 33 to ("adult (19 to 44 years)" or "middle age (45 to 64 years)" or "all aged (65 and over)")	5606

Table S3: Search strategy for Ovid Embase from 1990 to April 06, 2020, and updated on 10 February 2022

N	Search Terms	No. Of Identified Records
1	exp pregnancy complications/	120416
2	exp Hypertension, Pregnancy-Induced/	18081
3	exp Diabetes, Gestational/	34337
4	exp Abortion, Spontaneous/	38594
5	exp infant, low birth weight/	61045
6	exp infant, premature/	100234
7	exp Infant, Small for Gestational Age/	15230
8	exp Premature Birth/	100234
9	exp stillbirth/	16980
10	exp Fetal Death/	36885
11	exp Abruptio Placentae/	6167
12	exp Fetal Growth Retardation/	43158
13	exp cardiovascular disease/	3985963
14	exp heart diseases/	1797664
15	exp Atrial Fibrillation/	67073
16	exp arrhythmias/	457867
17	arrhythmi* or cardiac dysrhythmia* or atrial fibrillation or afib or a-fib or atrial flutter* or bradycardi* or heart block* or tachycardi* or Tachyarrhythmi* or ventricular fibrillation* or ventricular flutter*.ab,hw,kw,ti.	487004

18	Clinical study/	154917
19	case control study/	153820
20	Family study/	26014
21	Longitudinal study/	137625
22	Retrospective study/	898534
23	Prospective study/	591336
24	Randomized controlled trials/	176769
25	23 not 24	585133
26	Cohort analysis/	564690
27	(Case control adj (study or studies)).tw.	131504
28	(follow up adj (study or studies)).tw.	62568
29	(observational adj (study or studies)).tw.	162440
30	(epidemiologic$ adj (study or studies)).tw.	104996
31	(cross sectional adj (study or studies)).tw.	211389
32	1 or 2 or 3 or 4 or 5 or 6 or 7 or 8 or 9 or 10 or 11 or 12	322996
33	13 or 14 or 15 or 16 or 17	4029103
34	or/18-22,25-31	2606270
35	32 and 33 and 34	18264
36	limit 35 to (human and english language and yr="1990 - Current")	16858
37	limit 36 to (adult <18 to 64 years> or aged <65+ years>)	7989

Table S4. Baseline characteristics of the participants in the included studies.

**Characteristics**	Ray *et al* [2]	Leon *et al* [3]	Honigberg *et al* [4]	Yu *et al* [7]	Park *et al* [8]	Oliver-Williams *et al* [9]
**Race/ Ethnicity**	Not reported	White	Caucasian	Danish origin	Not reported	White
Exposed, n(%)	-	21,082 (82.5)	2,623 (93.4)	15,911 (75)	-	55,706 (74.8)
Non-exposed, n(%)	-	1,021,267 (79.9)	204,894 (94.3)	854,315 (87)	-	1,598,198 (72.7)
**Hypertension,** definition	ICD-9 and ICD 10-CA ≤ 1 year before the index birth date	ICD10 and Read codes or BP readings or medication before one year of the index pregnancy	ICD-9 and ICD-10 or self-reported at enrolment	Excluded	Excluded but BP reading before birth included Systolic BP, mean (SD)	Excluded
Exposed, n(%)	15,395 (20.5)	2,117 (8.3)	1,889 (67.3)	-	118.0 (13.9)	-
Non-exposed, n(%)	24,751 (2.3)	27,027 (2.1)	55,098 (25.4)	-	110.4 (11)	-
**Diabetes,** definition	ICD-9 and 10-CA before the index birth date	ICD10 and Read code	ICD 9-10 or self-reported at enrolment	Excluded	FBS, mg/dL reading before birth	ICD-10 using CALIBER algorithm before birth
Exposed, n(%)	6,974 (9.3)	358 (1.4)	177 (6.3)	-	89.5 (19.3)	527 (0.7)
Non-exposed, n(%)	56,034 (5.3)	3,020 (0.2)	8,828 (4.1)	-	86.8 (12.6)	3,886 (0.2)
**Obesity,** definition	ICD-9 and ICD-10 CA ≤ 1 year before index birth date	BMI measurement between 16 weeks' gestation and 5 years before the index pregnancy	BMI at enrolment, mean (SD)	Pre-pregnancy obesity (yes, no)	BMI before birth >30 kg/m2	Not reported
Exposed, n(%)	2,363 (3.1)	2,735(23.6)	28.1 (5.6)	3,918 (18)	7,784 (20.9)	-
Non-exposed, n(%)	15,263 (1.4)	47,284 (13)	27.1 (5.1)	37,267 (4)	157,526 (7.9)	-
**Dyslipidaemia,** definition	ICD-9 and 10-CA ≤ 1 year before the index birth date	Not reported	ICD 9-10 or self-reported at enrolment	Not reported	Total cholesterol, mg/dL reading before birth	Not reported
Exposed, n(%)	628 (0.8)	-	305 (10.9)	-	181.0 (32.7)	-

Non-exposed, n(%)	6,930 (0.7)	-	24,523 (11.3)	-	175.8 (33.6)	-
**Smoking,** definition	Drug dependence or smoking ICD-9 and ICD-10 CA ≤ 1 year before index birth date	ICD10 and read the code closest to the start of the index pregnancy	Not clear	Smoking during pregnancy (yes, no)	Current smoker	Not clear
Exposed, n(%)	1,015 (1.3)	5,361 (34.9)	928 (33.0)	3,278 (17)	1,810 (4.9)	1,595 (2.1)
Non-exposed, n(%)	11,111 (1.1)	193,893 (39.3)	88,399 (40.7)	123,130 (18)	76,931 (3.9)	47,232 (2.1)
**Multifetal**			Not reported	Not reported		Excluded
Exposed, n(%)	3,661 (4.9)	1,210 (6.00)	-	-	2244 (6.0)	-
Non-exposed, n(%)	16,719 (1.6)	29,261 (2.80)	-	-	30 041 (1.5)	-

CALIBER: Cardiovascular disease research using linked bespoke studies and electronic health records: FBS: Fasting Blood Sugar; BP: blood pressure; mg/dL: Milligrams per decilitre; SD: Standard deviation

Table S5. Risk of Bias Assessment in included Studies using Newcastle-Ottawa Scale.[1]

Study ID	Selection	Comparability	Outcome/ exposure	Overall stars	Overall Assessment*
Ray et al, 2012[2]	4	2	2	8	Good
Leon et al, 2019[3]	4	2	2	8	Good
Honigberg et al, 2019[4]	3	1	2	6	Good
Garovic et al, 2020[5]	4	2	3	9	Good
Auger et al, 2020[6]	4	2	3	9	Good
Yu et al, 2021[7]	4	2	3	9	Good
Park et al, 2022[8]	4	2	2	8	Good
Oliver-Williams *et al.,* 2022[9]	4	2	2	8	Good

*The scoring system as follows:

- Good quality: 3 or 4 stars in selection domain and 1 or 2 stars in comparability domain and 2 or 3 stars in outcome/exposure domain.
- Fair quality: 2 stars in selection domain and 1 or 2 stars in comparability domain and 2 or 3 stars in outcome/exposure domain.
- Poor quality: 0 or 1 star in selection domain OR 0 stars in comparability domain OR 0 or 1 stars in outcome/exposure domain.

References

1. Wells GA, Shea B, O'Connell Da, et al. The Newcastle-Ottawa Scale (NOS) for assessing the quality of nonrandomised studies in meta-analyses. In: Oxford; 2000.
2. Ray JG, Schull MJ, Kingdom JC, Vermeulen MJ. Heart failure and dysrhythmias after maternal placental syndromes: HAD MPS Study. *Heart.* 2012;98(15):1136-1141.
3. Leon LJ, McCarthy FP, Direk K, et al. Preeclampsia and Cardiovascular Disease in a Large UK Pregnancy Cohort of Linked Electronic Health Records: A CALIBER Study. *Circulation.* 2019;140(13):1050-1060.
4. Honigberg MC, Zekavat SM, Aragam K, et al. Long-Term Cardiovascular Risk in Women With Hypertension During Pregnancy. *J Am Coll Cardiol.* 2019;74(22):2743-2754.
5. Garovic VD, White WM, Vaughan L, et al. Incidence and Long-Term Outcomes of Hypertensive Disorders of Pregnancy. *J Am Coll Cardiol.* 2020;75(18):2323-2334.
6. Auger N, Potter BJ, He S, Healy-Profitos J, Schnitzer ME, Paradis G. Maternal Cardiovascular Disease 3 Decades After Preterm Birth: Longitudinal Cohort Study of Pregnancy Vascular Disorders. *Hypertension.* 2020;75(3):788-795.
7. Yu Y, Soohoo M, Sorensen HT, Li J, Arah OA. Gestational Diabetes Mellitus and the Risks of Overall and Type-Specific Cardiovascular Diseases: A Population- and Sibling-Matched Cohort Study. *Diabetes Care.* 2022;45(1):151-159.
8. Park Y, Cho GJ, Roh SY, Na JO, Oh MJ. Increased Cardiac Arrhythmia After Pregnancy-Induced Hypertension: A South Korean Nationwide Database Study. *J Am Heart Assoc.* 2022;11(2):e023013.
9. Oliver-Williams C, Stevens D, Payne RA, Wilkinson IB, Smith GCS, Wood A. Association between hypertensive disorders of pregnancy and later risk of cardiovascular outcomes. *BMC Med.* 2022;20(1):19.
